# Serum indoxyl sulfate is associated with mortality in hospital-acquired acute kidney injury: a prospective cohort study

**DOI:** 10.1186/s12882-019-1238-9

**Published:** 2019-02-14

**Authors:** Wenji Wang, Guihua Hao, Yu Pan, Shuai Ma, Tianye Yang, Peng Shi, Qiuyu Zhu, Yingxin Xie, Shaojun Ma, Qi Zhang, Hong Ruan, Feng Ding

**Affiliations:** 1 Division of Nephrology, Shanghai Ninth People’s Hospital, School of Medicine, Shanghai Jiaotong University, 639 Zhizaoju Road, Shanghai, 200011 China; 2 Department of Medical Statistics, Children’s Hospital; Center for Evidence-based Medicine, Fudan University, Shanghai, 200433 China; 3 Department of Nursing, Clinical Medical School, Shanghai Ninth People’s Hospital, School of Medicine, Shanghai Jiaotong University, 639 Zhizaoju Road, Shanghai, 200011 China

**Keywords:** Acute kidney injury, Protein-bound solute, Indoxyl sulfate, Prognosis

## Abstract

**Background:**

Protein-bound uremic toxins are associated with poor outcomes in patients with chronic kidney disease. The aim of this study is to investigate the relationship between indoxyl sulfate (IS), a protein-bound solute, and 90-day mortality in patients with acute kidney injury.

**Methods:**

Adults with hospital-acquired AKI (HA-AKI) were enrolled in this prospective cohort study between 2014 and 2015, according to the KDIGO creatinine criteria. The primary end point was all-cause death during follow-up.

**Results:**

The mean serum IS level in patients with HA-AKI was 2.74 ± 0.75 μg/ml, which was higher than that in healthy subjects (1.73 ± 0.11 μg/ml, *P* < 0.001) and critically ill patients (2.46 ± 0.35 μg/ml, *P* = 0.016) but was lower than that in patients with chronic kidney disease (3.07 ± 0.31 μg/ml, *P* < 0.001). Furthermore, serum IS levels (2.83 ± 0.55 μg/ml) remained elevated in patients with HA-AKI on the seventh day after AKI diagnosis. Patients with HA-AKI were divided into the following two groups according to the median serum IS level: the low-IS group and the high-IS group. A total of 94 (35.9%) patient deaths occurred within 90 days, including 76 (29.0%) in the low-IS group and 112 (42.7%) in the high-IS group (*P* = 0.019). Kaplan-Meier analysis revealed that the two groups differed significantly with respect to 90-day survival (log-rank *P* = 0.007), and Cox regression analysis showed that an IS level ≥ 2.74 μg/ml was significantly associated with a 2.0-fold increased risk of death (adjusted hazard ratio [HR], 2.92; 95% confidence interval [CI], 1.76 to 4.86; *P* < 0.001) compared with an IS level < 2.74 μg/ml.

**Conclusions:**

Serum IS levels were significantly elevated in patients with HA-AKI compared to those in healthy subjects and critically ill patients and were associated with a worse prognosis of HA-AKI.

**Trial registration:**

www.clinicaltrials.gov NCT 00953992. Registered 6 August 2009.

**Electronic supplementary material:**

The online version of this article (10.1186/s12882-019-1238-9) contains supplementary material, which is available to authorized users.

## Background

Acute kidney injury (AKI) is common in hospitalized patients and is associated with a high risk of adverse outcomes, especially in critically ill patients [1, 2]. Despite the diagnostic and therapeutic advancements in AKI, the AKI related mortality remains high [3, 4]. Moreover, a portion of patients with AKI suffer from progression of chronic kidney disease (CKD) even end stage kidney disease (ESKD) and is severe enough to raise morbidity and mortality rates.

Retention of various solutes, mainly excreted into urine by the kidneys in normal persons, is observed in patients with AKI. Solutes that affect biological functions are referred to as uremic toxins [5, 6]. Many studies have focused on the circulating concentrations of protein-bound uremic toxins, especially indoxyl sulfate (IS) and p-cresyl sulfate, and on linking these solutes to mortality and cardiovascular (CV) events in non-dialysis CKD and dialysis patients [7, 8]. IS is an organic anion converted from indole, which is the decomposition product of tryptophan by intestinal bacteria [9]. It is a representative protein-bound solute, and its deleterious effects of glomerular sclerosis and interstitial fibrosis have been studied in various cell types and in animal models [10]. IS has been shown to induce inflammation, oxidative stress, enhance collagen accumulation, and inhibit endothelial proliferation and wound repair, leading to tubular toxicity, interstitial fibrosis, glomerular sclerosis, and further renal function deterioration. IS exerts these effects by affecting the expression of various cytokines, such as intercellular adhesion molecule 1, plasminogen activator inhibitor 1, and transforming growth factor-beta 1 (TGF-β1) [11–14]. These findings have been reinforced by additional clinical studies. Higher levels of serum IS were found to be independently associated with CKD progression, all-cause mortality and CV mortality in patients with CKD [15–17].

IS has also been reported as a potential toxin in AKI models. The results in a study Vin-Cent Wu demonstrated that circulating IS was elevated in 41 patients with AKI and had direct effects on human endothelial progenitor cells via NO-dependent mechanisms in kidney arteriolar endothelium after kidney I/R injury in mice [18]. Another study showed that the concentration of IS in the serum, brain and kidney significantly increased 24-84 h after commencement of cisplatin treatment during AKI, thereby accelerating renal and central nervous system toxicity [19]. However, the data on IS levels in AKI are limited, and the relationship between IS and the prognosis of AKI is still unclear. We conducted this prospective cohort study to evaluate concentrations of serum IS and to investigate the association between IS levels and 90-day mortality in hospital-acquired AKI (HA-AKI) after controlling for comorbidities, other uremic toxins and nutritional markers.

## Methods

### Study population and definitions

Consecutive patients with HA-AKI, aged ≥18 years, were prospectively entered into the cohort from July 2014 to December 2015. Patients with preexisting chronic kidney failure, known acute renal dysfunction, rapidly progressive glomerulonephritis or post-renal obstruction as the main cause of AKI, hospital stays < 24 h, or malignancy were excluded. AKI was diagnosed using the creatinine criteria of 2012 Kidney Disease: Improving Global Outcomes (KDIGO) [20]. Chronic kidney failure was determined as an estimated glomerular filtration rate (eGFR) lower than 60 ml/min per 1.73 m^2^, which was calculated by applying the MDRD (Modification of Diet in Renal Disease) equation with appropriate adjustments for female patients [21]. This study adhered to the Declaration of Helsinki and was approved by the ethics committee of Shanghai Ninth People’s Hospital, School of Medicine, Shanghai Jiaotong University (approval number: [2014]45). Written informed consent was provided by all participants prior to their enrollment in the study, which was registered at www.clinicaltrials.gov (NCT 00953992). All protocols were implemented in accordance with the relevant guidelines and regulations.

### Data collection and blood sampling

Investigators monitored data of inpatients’ serum creatinine daily by the hospital information system (HIS). Patients with levels of serum creatinine that rose as specified by the KDIGO criteria within one week were evaluated by nephrologists within 24 h. All of the collected data were extracted from electronic medical records by the nephrologists and were entered into a computer form using EpiData software by the investigators. The information recorded included the following: demographic characteristics (gender, age, medical history, comorbid conditions, surgical history, cause of AKI), illness severity (evaluated using the non-renal Acute Physiology and Chronic Health Evaluation [APACHE] II score, derived from the APACHE II score minus renal parameters score, on the day of AKI diagnosis), and renal functional parameters (serum creatinine, urea nitrogen, AKI stage, renal replacement therapy [RRT]). Information pertaining to biochemical parameters, including serum cholesterol, alanine aminotransferase (ALT), albumin, high sensitivity C-reactive protein (hsCRP), hemoglobin, white blood cell counts, neutrophils percentage, red blood cell (RBC) counts and platelet counts, was also recorded. Blood samples were collected at baseline, at the time of AKI diagnosis (Diagnosis), and again on the seventh day after AKI diagnosis (Day7). A portion of each biological sample was evaluated immediately in the hospital laboratory, and the remainder of the sample was stored at − 80 °C for subsequent measurement of IS and β_2_-microglobulin levels.

### Biochemical measurements

Most biochemical parameters were measured in the hospital clinical laboratory with automated methods. The concentrations of hsCRP (Aristo, Goldsite, China) and β_2_-microglobulin (BN prospec system, Siemens, Germany) were measured using immunoturbidimetry assay, and total IS levels were quantified by HPLC with UV detection in a Model 1100 series liquid chromatograph (Agilent, CA, USA). The serum specimens were deproteinized, and bound IS was displaced by adding 300 ml of methanol to 80 ml of plasma. After being vortex-mixed vigorously, the samples were centrifuged at 8000×g for 15 min at 4 °C. Chromatographic separation was subsequently performed on a Kromasil column (Kromasil 100–3.5, C18, 150 × 2.1 mm, 5 μm) coupled to a guard column (10 × 2.1 mm, 5 μm), with 0.15% trifluoroacetic acid-H_2_O serving as mobile phase A (75% *v*/v), and methanol serving as mobile phase B (25% v/v). The mobile phase was delivered at a flow rate of 0.20 ml/min at 35 °C. Detection was performed at 280 nm by a UV detector. Acquired data were processed with ChemStation software (Version A.09. 01, Agilent, CA, USA). The method was found to be linear over a concentration range of 0.1–50.0 g/ml, and the correlation coefficient was more than 0.997. The range of recovery for IS was 93.8–97.3%. The intra- and inter-assay mean biases were less than 8.8 and 11.1%, respectively.

### Study protocol

Patients with HA-AKI enrolled in the study. They were followed prospectively from the time of the nephrologist consultation until their dates of death. The follow-up period of the study was 90 days, and the primary end point was all-cause mortality. Then we analyzed the data from patients with serum samples on Day7 to reveal the longitudinal changes in concentrations of serum IS over time and to compare these changes with those in serum creatinine and β_2_-microglobulin levels.

### Statistical analyses

On the basis of an assumed 90-day mortality of 30.0% for patients with lower levels of serum IS when AKI was diagnosed, a sample size of 322 patients was required for 80% power to detect a 10.0% difference in all-cause 90-day mortality (hazard ratio [HR] of 2.0, at the 5.0% level of significance) [22]. The mortalities of patients with lower and higher levels of serum IS and the HR are based on a preliminary study of small samples.

Continuous variables were expressed as means ± SDs or as medians (interquartile ranges), and categorical variables were expressed as frequencies (percentage). Comparisons of continuous and categorical data for patients in different groups were performed by independent samples *t* tests or the Kruskal-Wallis test and chi-squared tests, respectively, as appropriate. Paired *t* tests were used to examine the longitudinal changes in serum IS, creatinine or β_2_-microglobulin levels over time.

Kaplan-Meier analyses were used to assess the differences in surviving proportions between the IS, creatinine and β_2_-microglobulin subgroups. Cox proportional hazard models were performed to calculate the relative risks of all-cause death. Univariate Cox regression was performed to identify potential confounding variables, and the multivariable Cox regression model consisted of variables with a *P* value < 0.2 in the univariate Cox regression model. In the multivariable Cox model, we added all first-order interactions in each model and retained interaction terms with *P* < 0.1 in the final model to evaluate the effect of IS, creatinine or β2-microglobulin was independently associated with the hazard of all-cause death considering their interaction with another factors.

All *P* values were two tailed, and *P* values < 0.05 were considered significant. Statistical analyses were performed with SAS version 9.0 (SAS Inc., Cary, NC).

## Results

### Patient cohort

**Fig. 1:**
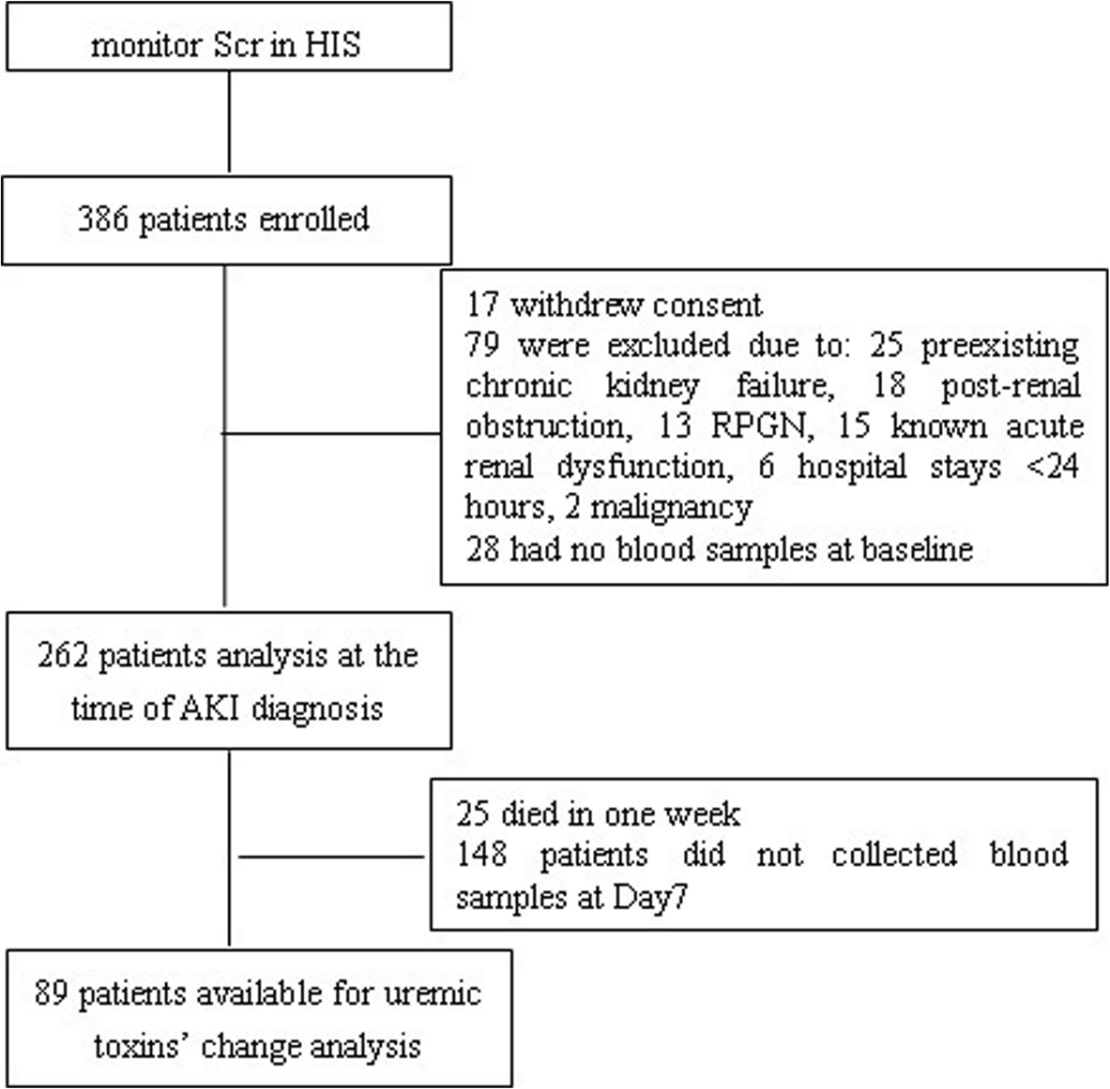
Flow chart of study progress. HIS, hospital information system; Scr, serum creatinine; RPGN, rapidly progressing glomerulonephritis

Of the 386 patients enrolled in the study, 79 patients were excluded, 17 patients withdrew consent, and 28 patients provided no blood samples at baseline. Consequently, 262 patients were followed for 90 days prospectively. A total of 148 patients did not provide serum samples on Day7, and 25 patients died within a week after being diagnosed with AKI. Thus, 89 patients were available for uremic toxins’ changes analysis (Fig. 1).

### Serum IS levels were elevated in patients with AKI

Serum IS levels were measured in the following four groups: 262 patients with AKI, 65 patients with CKD, 51 critically ill patients without AKI in ICU, and 65 healthy persons serving as normal controls. There were no significant differences in age and gender among the four groups, nor was there a significant difference in the levels of serum creatinine between the AKI and CKD groups or the APACHE II score between the AKI and critically ill groups. The average level of IS in patients with AKI at the time of AKI diagnosis was 2.7 ± 0.8 μg/ml, which was significantly higher than that in healthy people and critically ill patients (2.7 ± 0.8 μg/ml vs 1.7 ± 0.1 μg/ml, *P* = 0.001; 2.7 ± 0.8 μg/ml vs 2.5 ± 0.3 μg/ml, *P =* 0.03; respectively). However, the IS level in patients with AKI was lower than that in patients with CKD (2.7 ± 0.8 μg/ml vs 3.1 ± 0.3 μg/ml, *P <* 0.001).

### Clinical and biochemical characteristics of the AKI cohort

The cohort of 262 patients with AKI consisted of 195 men and 67 women, with a mean age of 62.6 ± 16.6 years (range, 18 to 97 years). The patients were categorized into the following two groups according to the median serum IS level (2.74 μg/ml), which was measured at the time of AKI diagnosis: a low-IS group (IS < 2.74 μg/ml, *n* = 131) and a high-IS group (IS ≥2.74 μg/ml, n = 131). Patients’ demographic characteristics, clinical features, laboratory measures, and in-hospital and 90-day mortality rates for two groups are shown in Table 1. Patients in the low-IS group were more likely to have a previous history of diabetes mellitus, underwent less operations before suffering AKI and needed less RRT than patients in the high-IS group, while patients in the high-IS group had higher serum creatinine, urea nitrogen, β_2_-microglobulin, and hemoglobin levels and higher RBC counts than patients in the low-IS group. There were no significant differences in other characteristics between the two groups.

**Table 1:** Demographic, clinical, laboratory values and mortality in total serum IS and according to the 2 a priori-selected groups of serum IS levels in 262 patients at the time of AKI diagnosis

	Total *n* = 262	IS < 2.74 μg/ml *n* = 131	IS ≥ 2.74 μg/ml *n* = 131	*P*
In-hospital mortality, *n* %	73 (27.9)	26 (19.8)	47 (35.9)	0.004
90d mortality, *n* %	94 (35.9)	38 (29.0)	56 (42.7)	0.019
Demographic				
Age, yr	62.6 ± 16.6	64.04 ± 15.29	61.11 ± 17.78	0.154
Male, *n* %	195 (74.4)	96 (73.3)	99 (75.6)	0.389
MAP, mmHg	85.3 (73.3, 96.7)	86.7 (74.8, 98.5)	84.2 (68.5, 94.5)	0.073
Comorbid conditions, *n* %				
Hypertension	102 (38.9)	51 (38.9)	51 (38.9)	0.550
Coronary heart disease	42 (16.0)	24 (18.3)	18 (13.7)	0.312
Diabetes mellitus	46 (17.5)	29 (22.1)	17 (13.0)	0.037
Chronic hepatic disease	13 (5.0)	8 (6.1)	5 (3.8)	0.286
Chronic kidney disease	20 (7)	7 (5.3)	13 (9.9)	0.122
Surgery	187 (71.4)	81 (61.8)	106 (80.9)	< 0.001
Sepsis	76 (29.0)	33 (25.2)	43 (32.8)	0.110
AKI stage at diagnosis, *n* %				
1	119 (45.5)	64 (48.9)	55 (42.0)	0.264
2	63 (24.0)	29 (22.1)	34 (26.0)	0.470
3	80 (30.5)	38 (29.0)	42 (32.0)	0.591
RRT, *n* %	40 (15.3)	13 (9.9)	27 (20.6)	0.016
Mechanical ventilation, *n* %	96 (36.6)	42 (32.1)	54 (41.2)	0.079
APACHE II score	18.4 ± 8.7	17.5 ± 8.6	19.4 ± 8.9	0.117
Biochemical measurements				
Serum				
Creatinine, μmol/L	167 (137,226)	153 (132,195)	177 (146,265)	0.001
Urea nitrogen, mmol/L	15 (11.1,21.9)	13.9 (9.5,20.6)	17.1 (12.5,25.1)	0.001
β2-microglobulin, mg/L	5.1 (3.6,8.2)	4.3 (2.9, 6.6)	6.3 (4.2, 8.6)	0.001
Albumin, g/L	32.7 ± 7.1	32.8 ± 7.3	29.2 ± 7.6	0.095
Cholesterol, mmol/L	6.3 (4.1, 10.1)	7.5 (4.6,11.4)	5.1 (3.9, 8.8)	0.063
ALT, IU/L	31.5 (19.0,62.8)	27.0 (19.0, 57.8)	35.0 (19.3, 70.8)	0.186
hsCRP, mg/L	95.3 ± 76.3	85.6 ± 81.5	107.4 ± 68.0	0.095
Blood				
WBC counts, × 109 cell/L	13.4 ± 6.8	13.1 ± 8.0	13.8 ± 5.4	0.389
Neutrophilic granulocyte, %	79.4 (16.2,86.9)	79.9 (17.4,85.8)	77.9 (14.8,88.1)	0.588
RBC counts, × 1012 cell/L	3.6 ± 0.8	3.5 ± 0.8	3.7 ± 0.9	0.045
Hemoglobin, g/L	108.3 ± 24.0	104.6 ± 21.5	112.0 ± 25.9	0.013
Platelet counts, ×1012/L	150.4 ± 92.1	160.1 ± 100.0	140.5 ± 82.4	0.085

*IS* indoxyl sulfate, *AKI*, acute kidney injury, *MAP* mean arterial pressure, *RRT* renal replacement therapy, *ALT* alanine aminotransferase, *hsCRP* high sensitivity C-reactive protein, *WBC* white blood cell, *RBC* red blood cell

### Serum IS levels were associated with 90-day mortality

**Fig. 2:**
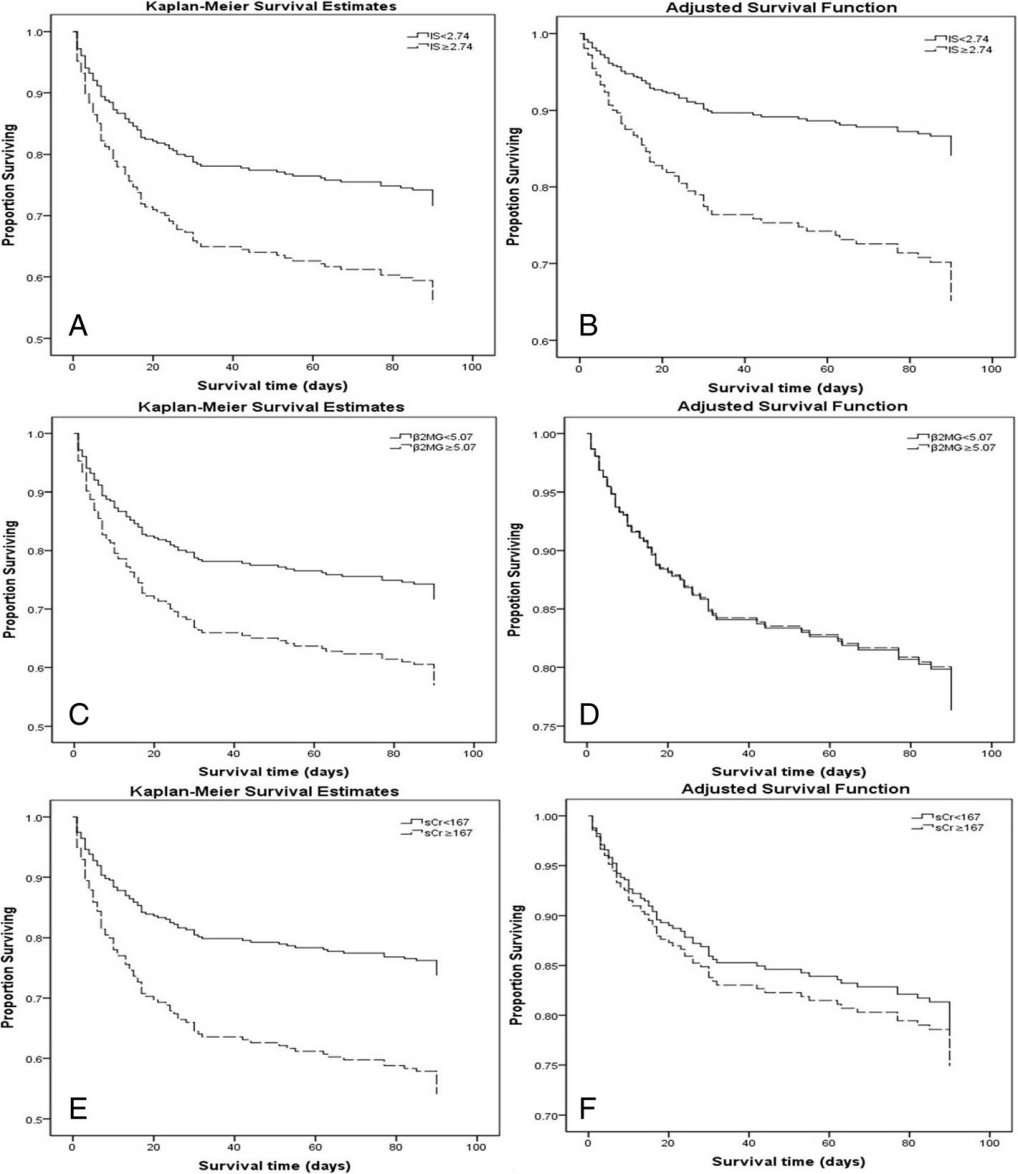
Kaplan-Meier proportion of surviving patients after 90 days of observation according to the 2 a priori-selected groups of uremic toxins in 262 patients occurring AKI. (**a**) IS, unadjusted; (**b**) IS, adjusted for age, gender, non-renal APACHE II score, sepsis, RRT, surgery, serum albumin, creatinine, BUN, β2-microglobulin, ALT, neutrophilic granulocyte and platelet counts; (**c**) β2-microglobulin, unadjusted; (**d**) β2-microglobulin, adjusted for age, gender, non-renal APACHE II score, sepsis, RRT, surgery, serum albumin, IS, creatinine, BUN, ALT, neutrophilic granulocyte and platelet counts; (**e**) Creatinine, unadjusted; (**f**) Creatinine, adjusted for age, gender, non-renal APACHE II score, sepsis, RRT, surgery, serum albumin, IS, BUN, β2-microglobulin, ALT, neutrophilic granulocyte and platelet counts. The cutoff point for serum IS is 2.74 μg/ml, while that for serum creatinine is 167 μmol/L, and that for β2-microglobulin is 5.07 mg/L

The overall in-hospital and 90-day mortality in 262 patients were 27.9 and 35.9%, respectively. Both in-hospital mortality and 90-day mortality were significantly elevated in the high-IS group (35.9% vs 19.8%, *P* = 0.004 and 42.7% vs 29.0%, *P* = 0.019, respectively). The Kaplan-Meier survival curves for 90-day survival, stratified according to serum IS, creatinine and β_2_-microglobulin levels, are displayed in Fig. 2. There were significant differences in 90-day survival between the two IS groups, both in the unadjusted model and in the full-adjusted model. However, the differences in 90-day survival between the two serum creatinine groups (categorized by the median serum creatinine level of 167 μmol/L) or the two β_2_-microglobin groups (categorized by the median serum β_2_-microglobin level of 5.07 mg/L) were reduced in the full-adjusted models.

Various risk factors are related to all-cause death in patients with AKI. The results of univariate Cox regression showed that serum IS (hazard ratio [HR], 1.47; 95% confidence interval [95% CI], 1.12 to 1.93; *P* = 0.006) and β_2_-microglobulin (HR, 1.04; 95% C, 1.00 to 1.07; *P* = 0.041) were associated with 90-day all-cause mortality. Other variables that displayed associations with 90-day all-cause mortality included surgery before AKI, sepsis, renal replacement therapy, non-renal APACHE II scores, neutrophilic granulocyte counts, platelet counts, and ALT levels (Additional file 1: Table S1). Patients in the high-IS group had up to a 1.0-to-2.0-fold increased risk of death than patients in the low-IS group when IS was entered either as a dichotomous (HR, 2.92; 95% CI, 1.76 to 4.86; *P* < 0.001) or as a continuous (HR, 1.74; 95% CI, 1.27 to 2.39; *P* = 0.001) variable, as demonstrated by Cox regression after adjusted multivariate analysis (Table 2). However, increasing serum creatinine and β_2_-microglobin concentrations were not significantly associated with higher 90-day mortality after adjustment for the above confounding variables (Tables 3 and 4).

**Table 2:** Multivariate Cox proportional hazard model of mortality during 90-day follow-up in AKI patients (IS entered as a continuous or dichotomous variable)

Model	HR	95% CI	*P*
IS (continuous variable)			
Unadjusted	1.47	1.12–1.93	0.006
Model 1	1.48	1.11–1.96	0.007
Model 2	1.51	1.15–2.00	0.003
Model 3	1.47	1.11–1.97	0.008
Model 4	1.61	1.19–2.17	0.002
Model 5	1.74	1.27–2.39	0.001
IS (dichotomous variable)			
Unadjusted	1.75	1.16–2.64	0.008
Model 1	1.75	1.18–2.70	0.006
Model 2	2.01	1.30–3.10	0.002
Model 3	2.00	1.28–3.14	0.002
Model 4	2.47	1.53–3.98	< 0.001
Model 5	2.92	1.76–4.86	< 0.001

Variables of model 1 include age and gender

Variables of model 2 include age, gender, non-renal APACHE II score, sepsis, RRT, and surgery

Variables of model 3 include age, gender, non-renal APACHE II score, sepsis, RRT, surgery, serum albumin, creatinine, BUN and β_2_-microglobulin

Variables of model 4 include age, gender, non-renal APACHE II score, sepsis, RRT, surgery, serum albumin, creatinine, BUN, β_2_-microglobulin, ALT, neutrophilic granulocyte and platelet counts

Variables of model 5 include age, gender, non-renal APACHE II score, sepsis, RRT, surgery, serum albumin, creatinine, BUN, β2-microglobulin, ALT, neutrophilic granulocyte, platelet counts, and interaction terms (gender*platelet counts, surgery*serum albumin, surgery*creatinine and ALT*platelet counts)

**Table 3:** Multivariate Cox proportional hazard model of mortality during 90-day follow-up in AKI patients (β_2_-microglobulin entered as a continuous or dichotomous variable)

Model	HR	95% CI	*P*
β_2_-microglobulin (continuous variable)			
Unadjusted	1.03	1.00–1.07	0.041
Model 1	1.03	1.00–1.07	0.056
Model 2	1.00	0.96–1.04	0.929
Model 3	1.00	0.95–1.03	0.590
Model 4	1.01	0.96–1.05	0.813
Model 5	1.01	0.96–1.05	0.800
β_2_-microglobulin(dichotomous variable)			
Unadjusted	1.69	1.11–2.57	0.014
Model 1	1.67	1.10–2.53	0.017
Model 2	1.01	0.65–1.57	0.970
Model 3	0.88	0.55–1.40	0.581
Model 4	1.00	0.61–1.61	0.966
Model 5	1.00	0.61–1.67	0.987

Variables of model 1 include age and gender

Variables of model 2 include age, gender, non-renal APACHE II score, sepsis, RRT, and surgery

Variables of model 3 include age, gender, non-renal APACHE II score, sepsis, RRT, surgery, serum albumin, IS, creatinine and BUN

Variables of model 4 include age, gender, non-renal APACHE II score, sepsis, RRT, surgery, serum albumin, IS, creatinine, BUN, ALT, neutrophilic granulocyte and platelet counts

Variables of model 5 include age, gender, non-renal APACHE II score, sepsis, RRT, surgery, serum albumin, IS, BUN, creatinine, ALT, neutrophilic granulocyte, platelet counts and interaction terms (gender*platelet counts, surgery*serum albumin, surgery*creatinine, and ALT*platelet counts)

**Table 4:** Multivariate Cox proportional hazard model of mortality during 90-day follow-up in AKI patients (serum creatinine entered as a continuous or dichotomous variable)

Model	HR	95% CI	*P*
Serum creatinine (continuous variable)			
Unadjusted	1.00	1.00–1.00	0.002
Model 1	1.00	1.00–1.00	0.002
Model 2	1.00	1.00–1.00	0.696
Model 3	1.00	1.00–1.00	0.597
Model 4	1.00	1.00–1.00	0.924
Model 5	1.00	1.00–1.00	0.360
Serum creatinine (dichotomous variable)			
Unadjusted	2.02	1.32–3.07	0.001
Model 1	1.96	1.28–2.99	0.002
Model 2	1.07	0.68–1.68	0.762
Model 3	0.87	0.54–1.42	0.583
Model 4	1.14	0.69–1.88	0.608
Model 5	0.93	0.55–1.57	0.791

Variables of model 1 include age and gender

Variables of model 2 include age, gender, non-renal APACHE II score, sepsis, RRT, and surgery

Variables of model 3 include age, gender, non-renal APACHE II score, sepsis, RRT, surgery, serum albumin, IS, BUN and β_2_-microglobulin

Variables of model 4 include age, gender, non-renal APACHE II score, sepsis, RRT, surgery, serum albumin, IS, BUN, β_2_-microglobulin, ALT, neutrophilic granulocyte and platelet counts

Variables of model 5 include age, gender, non-renal APACHE II score, sepsis, RRT, surgery, serum albumin, IS, BUN, β2-microglobulin, ALT, neutrophilic granulocyte, platelet counts and interaction terms (gender*platelet counts, surgery*serum albumin, surgery*creatinine, and ALT*platelet counts)

### Changes in serum IS, creatinine and β_2_-microglobin levels over one week in AKI

**Fig. 3:**
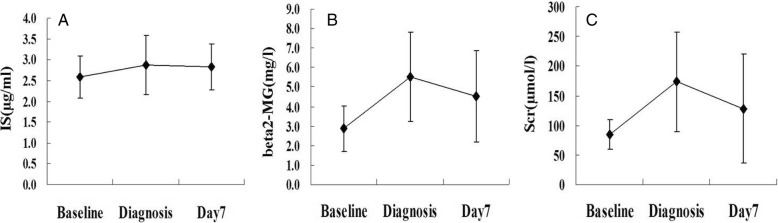
The changes of concentrations of uremic toxins in 89 patients occurring AKI. (**a**) serum indoxyl sulfate (IS); (**b**) serum β2-microglobulin (beta2-MG); (**c**) serum creatinine (Scr)

The levels of serum IS, creatinine and β_2_-microglobin were all significantly increased when AKI was diagnosed (2.88 ± 0.70 μg/ml vs 2.59 ± 0.51 μg/ml, *P* < 0.001; 173.5 ± 83.8 μmol/L vs 85.0 ± 25.2 μmol/L, *P* < 0.001; 5.52 ± 2.28 mg/L vs 2.86 ± 1.18 mg/L, *P* < 0.001; respectively) compared to baseline. To further investigate whether the longitudinal changes in serum IS levels were concordant with those in serum creatinine or β_2_-microglobin levels, their concentrations were remeasured on Day7 in 89 of the 262 patients with AKI. The results showed that serum IS levels remained high at Day7 (Day7 vs diagnosis: 2.83 ± 0.55 μg/ml vs 2.88 ± 0.70 μg/ml, *P* = 0.515), while levels of serum creatinine (128.1 ± 92.0 μmol/L vs 173.5 ± 83.8 μmol/L, *P* < 0.001) and β_2_-microglobin levels (4.52 ± 2.32 mg/L vs 5.52 ± 2.28 mg/L, *P* = 0.022) declined significantly (Fig. 3).

## Discussion

This prospective cohort study found that levels of serum IS were significantly increased at the time of AKI diagnosis compared to baseline and that serum IS levels were higher in patients with AKI than in critically ill patients with similar APACHE II scores but lower in patients with AKI than in patients with ESKD with similar eGFRs. Furthermore, serum IS levels remained high at one week after AKI was diagnosed, while serum creatinine and β_2_-microglobin levels decreased. Of note, the fully adjusted multivariable model showed that higher serum IS levels were correlated with an elevated risk of death within 90 days after AKI diagnosis.

According to the physicochemical characteristics affecting their clearance by dialysis, uremic toxins are mainly classified into three groups: small water-soluble molecules (such as creatinine), larger middle molecules (such as β_2_-microglobin), and protein-bound compounds (such as IS) [23]. Previous studies have focused on the circulating concentrations of uremic toxins in predialysis and dialysis patients because their biological activities have the capacity to damage almost every organ system [24]. However, there are limited published data on uremic toxins other than serum creatinine in AKI. Herrero-Morín et al. showed that the mean value of serum β_2_-microglobulin was 2.6 mg/L in critically ill children with a creatinine clearance (CrC) of less than 80 ml/minute per 1.73 m^2^ and that the correlation between the inverse of serum β_2_-microglobulin and CrC (*r* = 0.477) was stronger than the correlation between the inverse of creatinine and CrC (*r* = 0.104) [25]. Sahsivar et al. showed that serum β_2_-microglobulin levels were unchanged in a renal I/R rat model [26]. Serum IS levels were found to increase 10-to-15-fold with the progression of ischemia-induced AKI due to the downregulation of organic anion transporters (OATs) in the renal tubules [27] and to increase markedly 24–84 h after the commencement of cisplatin treatment [19]. In this study, the mean serum IS and β_2_-microglobulin levels, 2.7 μg/ml and 5.1 mg/l, respectively, were both significantly increased when AKI was diagnosed compared with baseline.

Moreover, the results showed that serum IS levels remained elevated, while serum creatinine and β_2_-microglobulin levels decreased rapidly within one week after AKI diagnosis. The persistently high IS levels in AKI may be mainly caused by abnormal OAT activity in tubular cells and gut microbe dysbiosis. AKI is characterized by tubular dysfunction, which will affect IS excretion because the secretion of IS is performed mostly by OATs localized at basolateral membranes of proximal tubular cells rather than by glomerular filtration [27, 28]. Matsuzaki T et al. and Saigo C et al. demonstrated that IS levels were elevated with the reduction of OAT activity and that expression of mRNA and protein levels of both rOAT1 and rOAT3 was downregulated in rats with I/R-induced acute renal injury [27, 29]. In addition, previous studies showed that recovery of tubular function was delayed relative to glomerular filcration during AKI [30]. The persistent elevated serum IS on Day7, when serum creatine declined, suggested that the expression and function of OATs might not return to their normal levels when glomerular filtration improved. On the other hand, IS is absorbed and metabolized from indole, which is derived from the amino acid tryptophan by tryptophanase via intestinal bacteria [11]. Quantitative and qualitative alterations in intestinal microbiota and disruptions of gut barrier function have been noted in patients with kidney disease or critically ill patients [31, 32]. Secretion of urea into the intestinal tract due to loss of kidney function results in the accumulation of ammonia, which may influence the growth of commensal bacteria [33]. The accumulation of ammonia and the alteration in gut microbiota may lead to increased production of serum IS.

Given that IS is involved in CKD progression and is related to clinical outcome parameters in CKD, including in patients on hemodialysis and those undergoing kidney transplantation [15, 34–36], serum IS may be independently associated with a high risk of all-cause death in AKI. Furthermore, recent studies demonstrated that the increased mortality was not only due to AKI itself and the direct renal complications of AKI but induced by remote nonrenal organ dysfunctions also [37]. IS with biologic effects on glomerular and tubular injury, and distant nonrenal organ injury in AKI as well, would impact mortality and renal recovery in AKI.

In vitro and vivo studies showed that IS accumulats in renal tissue after I/R AKI and induces free radical [27], ROS production, and release of many cytokines and chemokines, such as monocyte chemotactic protein-1 and EGF receptor, leading to impairment of proximal tubular and endothelial cell function through ERK and JNK pathways [38–40]. IS also induces ER stress in proximal tubular cells and inhibits cell proliferation via two pathways downstream of ER stress, increased expression of matrix metalloproteinases [41]. The dysfunction of tubular cells and endothelial cells can finally cause tubulointerstitial fibrosis and glomerularscleosis. In addition, previous studies revealed that IS is remarkablely associated with risk factors of cardiac dysfunction and atherosclerosis [34, 42] through various mechanisms including impairment of endothelial cell repair, worsening of atherosclerotic lesions and increasing tissue factor expression in endothelial cells [43]. IS induces oxidative stress in endothelial cells by increasing NADPH oxidase activity and decreasing glutathione levels [44] and has direct effects on endothelial progenitor cells via NO-dependent mechanisms, resulting in impairing the endothelial healing ability and vascular regeneration in AKI mice [18]. Yabuuchi N et al. examined the role of IS in the dysregulation of aquaporin 5, a pulmonary predominant water channel, in the bilateral nephrectomy induced AKI model [45]. The results showed the accumulation of IS and thickening of interstitial tissue in lungs, which suggested the role of IS as a mediator involved in renopulmonary crosstalk in AKI. Evaluated IS in plasma and brain could also weaken the ability of the brain transporters to remove other toxins and was associated with central nervous system dysfunction [19].

The renal and endothelial injuries, as well as non-renal organ dysfunction, observed in pre-clinical studies indicated that IS may lead to progression of kidney disease and cardio-vascular outcomes resulting increased mortality. An increased risk of progression of kidney disease in patients with higher total IS levels was found in Wu IW’s study, in which the progression defined as a 50% reduction in eGFR or dialysis initiation [15]. Barreto FC et al. demonstrated a higher IS level as a powerful predictor of vascular calcification, overall mortality and CV mortality. In their study, each 0.1-mg/ml increment in the IS serum level resulted in a 5% increase in the risk of death [16]. A recent study confirmed the association between elevated total IS levels and all-cause mortality with HR 1.30, but elevated p-cresol sulfate, DMA or MMA levels were not associated with mortality [17]. However, data on the relationship between IS and the prognosis in AKI are limited. Our observations showed that higher IS levels increased the risk of 90 day-mortality by up to 2.0-fold after adjustment for known risk factors of all-cause death in HA-AKI. Higher creatinine and β_2_-microglobulin levels were not associated with the risk of all-cause death in multivariable models. Therefore, increases in serum IS concentrations, which remained high after AKI was diagnosed, may more accurately reflect organ damage, including kidney and heart damage.

There are some limitations in this study. Patients in the study were obtained from a single center. Thus, selection bias during enrollment was inavoidable and we used multivariate analysis to adjust for comorbidities, other uremic toxins and nutritional markers. Most of the patients enrolled in this study were men, but there was no difference among the groups (*P* = 0.389). It was difficult to establish a link between IS and different causes of death due to absence of the cause of death. In addition, data regarding urine output were not available in this study. The incidence of AKI may have been underestimated, and the estimations of the AKI stages may have been affected, since urine output is an important criterion for the diagnosis of AKI. Additionally, IS excretion in urine could not be evaluated. IS is derived from dietary protein; thus, serum IS concentrations may be influenced by dietary protein intake. This study showed that urea nitrogen and serum creatinine levels were significantly higher in the high-IS group than in the low-IS group; however, albumin and cholesterol levels were similar between the two groups, but nPNA was not calculated in the study.

## Conclusions

This study confirmed that serum IS levels are high in patients with AKI and revealed that a higher level of serum IS was independently associated with a high risk of all-cause death. This association remained after adjustment for confounding risk factors. The results may give rise to the development of useful strategies for identifying patients at high risk for death. Clinical studies with larger sample sizes, longer follow-up time and certain causes of death as their end points are required to determine the exact correlations between serum IS levels and outcomes. Further study is also needed to reveal the mechanism underlying the relationships between serum IS levels and outcomes.

## Additional file

Additional file 1: **Table S1.** Univariate Cox proportional hazard model of mortality during 90-day follow-up in AKI patients. (PDF 247 kb)

## Abbreviations

AKI: Acute kidney injury
ALT: Alanine aminotransferase
APACHE: Acute physiology and chronic health evaluation
CKD: Chronic kidney disease
CrC: Creatinine clearance
CV: Cardiovascular
eGFR: Estimated glomerular filtration rate
ESKD: End stage kidney disease
HA-AKI: Hospital-acquired acute kidney injury
HIS: Hospital information system
HR: Hazard ratio
hsCRP: High sensitivity C-reactive protein
IS: Indoxyl sulfate
KDIGO: Kidney Disease: Improving Global Outcomes
MDRD: Modification of diet in renal disease
OATs: Organic anion transporters
RBC: Red blood cell
RRT: Renal replacement therapy
SIRS: Systemic inflammatory response syndrome
TGF-β1: Transforming growth factor-beta 1
