# Supplementary material for: Serum indoxyl sulfate is associated with mortality in hospital-acquired acute kidney injury: a prospective cohort study
Source: BMC Nephrol. 2019 Feb 14;20:57. doi: 10.1186/s12882-019-1238-9 (PMC6376694; doi:10.1186/s12882-019-1238-9)
Supplement: Supplementary file 1 — Table S1. Univariate Cox proportional hazard model of mortality during 90-day follow-up in AKI patients. (PDF 247 kb) [file 12882_2019_1238_MOESM1_ESM.pdf]

Supplemental Table. Univariate Cox proportional hazard model of mortality during 90-day follow-up in AKI patients

Variable	HR	95% CI	*P*
Age (yr)	1.002	0.990-1.015	0.450
Gender	1.682	0.991-2.853	0.054
MAP (mmHg)	1.006	0.999-1.013	0.111
History of diabetes	1.014	0.599-1.714	0.960
History of CHD	0.553	2.287-1.066	0.077
History of CKD	1.067	0.517-2.201	0.861
Surgery	0.319	0.204-0.500	**<0.001**
Sepsis	2.175	1.444-3.276	**<0.001**
RRT	2.032	1.259-3.278	**0.004**
Non-renal APACHE II score	1.128	1.101-1.156	**<0.001**
Creatinine (μmol/L)	1.001	1.000-1.002	**0.002**
BUN (mmol/L)	1.022	1.014-1.030	**<0.001**
IS (μg/mL)	1.470	1.116-1.934	**0.006**
β_2-MG (mg/L)	1.034	1.001-1.068	**0.041**
WBC counts ($\times 10^9$/L)	1.022	0.997-1.047	0.083
Neutrophilic granulocyte (%)	1.024	1.015-1.034	**<0.001**
RBC counts ($\times 10^{12}$/L)	1.073	0.820-1.404	0.607
Hemoglobin (g/dL)	1.002	0.992-1.011	0.280
Platelet counts ($\times 10^{12}$/L)	0.997	0.994-1.000	**0.026**
Serum albumin (g/dL)	0.974	0.946-1.003	0.083
ALT (U/L)	1.000	1.000-1.001	**<0.001**
hsCRP (mg/L)	1.002	0.998-1.006	0.278

AKI, acute kidney injury; MAP, mean arterial pressure; CHD, coronary heart disease; CKD, chronic kidney disease; RRT, renal replacement therapy; ALT, alanine aminotransferase; hsCRP, high sensitivity C-reactive protein; WBC, white blood cell
